# Changes in the Molecular Characteristics of Bovine and Marine Collagen in the Presence of Proteolytic Enzymes as a Stage Used in Scaffold Formation

**DOI:** 10.3390/md19090502

**Published:** 2021-09-02

**Authors:** Marfa N. Egorikhina, Ludmila L. Semenycheva, Victoria O. Chasova, Irina I. Bronnikova, Yulia P. Rubtsova, Evgeniy A. Zakharychev, Diana Ya. Aleynik

**Affiliations:** 1 Federal State Budgetary Educational Institution of Higher Education, Privolzhsky Research Medical University, the Ministry of Health of the Russian Federation (FSBEI HE PRMU MOH), Minin and Pozharsky Square 10/1, 603950 Nizhny Novgorod, Russia; bronnikova_i@pimunn.net (I.I.B.); rubcova_yup@pimunn.net (Y.P.R.); alejnik_d@pimunn.net (D.Y.A.); 2 Faculty of Chemistry, Lobachevsky State University of Nizhny Novgorod, pr. Gagarina 23, 603950 Nizhny Novgorod, Russia; semenycheva@ichem.unn.ru (L.L.S.); chasova@ichem.unn.ru (V.O.C.); eazakh@ichem.unn.ru (E.A.Z.)

**Keywords:** scaffold, biopolymers, collagen, fibrinogen, hydrolysis, thrombin, pancreatin

## Abstract

Biopolymers, in particular collagen and fibrinogen, are the leading materials for use in tissue engineering. When developing technology for scaffold formation, it is important to understand the properties of the source materials as well as the mechanisms that determine the formation of the scaffold structures. Both factors influence the properties of scaffolds to a great extent. Our present work aimed to identify the features of the molecular characteristics of collagens of different species origin and the changes they undergo during the enzymatic hydrolysis used for the process of scaffold formation. For this study, we used the methods of gel-penetrating chromatography, dynamic light scattering, reading IR spectra, and scanning electron microscopy. It was found that cod collagen (CC) and bovine collagen (BC) have different initial molecular weight parameters, and that, during hydrolysis, the majority of either type of protein is hydrolyzed by the proteolytic enzymes within the first minute. The differently sourced collagen samples were also hydrolyzed with the formation of two low molecular fractions: Mw ~ 10 kDa and ~20 kDa. In the case of CC, the microstructure of the final scaffolds contained denser, closely spaced fibrillar areas, while the BC-sourced scaffolds had narrow, short fibrils composed of unbound fibers of hydrolyzed collagen in their structure.

## 1. Introduction

The issue of restoring the integrity and functional adequacy of damaged or lost tissues is one of the most pressing challenges in modern medicine. At present, it is inextricably linked with the task of creating new materials for regenerative medicine. Such new biomimetic materials require certain universal properties (lack of toxicity, biocompatibility, low immunogenicity, etc.), as well as the ability to simulate the individual properties that determine the original tissue-specific features of the material being repaired (biodegradation rate, porosity, mechanical integrity, elasticity, etc.) while providing low-cost, minimally labor-intensive solutions. One of the main properties required of materials for tissue engineering and of the scaffolds based on them is good regenerative potential. This is provided by the biological activity of the material and its structural properties.

The biological activity that can be attained, and the structure of the material or scaffold that can be generated, strongly depend on the components from which they have been formed. Therefore, it is extremely important to be aware of the required properties of the material being developed as early as at the stage of selecting its constituents. Collagen is a recognized leader among components for creating biomaterials, scaffolds, and tissue-engineered constructs. It is known for its intense biological activity, which is preserved during the formation of various materials, scaffolds, inks for bioprinters, etc. [1,2,3,4]. The unique properties of this protein make it possible to create biomimetic constructs similar in structure to natural extracellular matrices [5,6,7]. The resulting materials/scaffolds have a porosity and microfiber structure that enables them to provide a large surface area for cell attachment and appropriate conditions for maintaining their viability, migration, and proliferation [8,9,10]. This protein blends perfectly with other materials, providing the possibility for creating hybrid or polycomposite scaffolds with improved properties compared to monocomposite constructs [11,12,13]. In particular, the combination of collagen and fibrin makes it possible to obtain hybrid scaffolds with mechanical characteristics that differ from those made purely of collagen or fibrin [14,15]. For example, the creation of collagen-based scaffold materials is carried out by introducing fragments of synthetic polymers into their composition, which contribute to the formation of the necessary spatial–geometric structures [16,17,18,19]. It is known, for example, that scaffolds based on collagen and acrylates possess improved mechanical properties and cytocompatibility [20,21,22,23,24,25]. It should also be taken into account that collagen is a thermally unstable polymer; at temperatures above 30–40 °C, its denaturation with the formation of gelatin begins [26,27,28,29]. Comparative data on the properties of hybrid materials based on collagen and gelatin proves the perspective of their use in combination or replacement of collagen with gelatin in scaffold-based technologies [30,31,32].

Early on, various types of collagen were isolated and described. They were shown to differ in their amino acid sequences and degree of modification—the extent of hydroxylation or glycosylation [33,34]. However, only in recent years have works begun to appear in which collagens belonging to the same type have been shown to demonstrate different properties depending on the species of animal from whose tissues they have been isolated [35,36,37,38]. A.M. Carvalho et al. [39] set forth a comparative study of type I collagen isolated from bovine skin, cod skin, and rat tails. The authors proved that, depending on their origin, the collagen samples varied in their amino acid composition, exhibited distinctive features in infrared spectra, and had dissimilar denaturation temperatures. The α-chains of the cod collagen had higher electrophoretic mobility compared with those of the mammalian collagens, which was due to their having a lower molecular weight. According to M. Gauza-Włodarczyk [40], certain differences were also established between fish collagen and cattle collagen when comparing their thermal properties. It is notable that analysis, using a bioinformatic analysis, of collagen from five sources of various origins revealed their differences, these being most pronounced between phylogenetically distant species, for example, between pigs and fish [41]. That is, the nature of the substrate from which the biopolymer is isolated largely determines its properties. Few data have appeared in the literature attesting that the nature of the collagen used can determine the properties of scaffolds. Therefore, A. Sorushanova et al. [42] showed that the properties of collagen sponges correlated with the origin of the collagen (from pig skin or bovine skin). Bovine collagen sponges had a larger pore diameter compared to sponges derived from pig collagen using the same technology. The bovine collagen sponges were more resistant to enzymatic denaturation and had a higher absorption capacity and elastic modulus. Another work has established that sheep collagen scaffolds have significantly higher ultimate strain and stress resistance and strength compared to scaffolds derived from cattle or pig collagen. They also appear to be more resistant to collagenase degradation compared to the cattle collagen samples, which, themselves, show a higher resistance than pig collagen scaffolds [43].

In our early works, it was established that hybrid hydrogel scaffolds formed from either cod collagen (CC) or bovine collagen (BC) in combination with fibrinogen (Fg) under the same conditions varied in their structural and mechanical properties [44]. Thus, it was shown that scaffolds made using BC had a denser structure compared to scaffolds made using CC. Differences in the elastic properties were also revealed. In particular, the compressive stress at a deformation value of 50% for the scaffold samples made using BC was 1.6 times higher than that for scaffold samples made with CC. It was also established that scaffolds made with CC and BC both had good biocompatibility and provided conditions that enabled stem cells to be maintained in a viable state, additionally sustaining their three-dimensional growth [44].

The technology for the formation of these scaffolds is based on enzymatic hydrolysis of the proteins [45]. The use of proteolytic enzymes, in the presence of which the spatial structure of scaffolds are formed from fragments of the protein hydrolysates, is not unique and is in widespread use in work with biopolymers [19,46,47,48,49]. Despite this, many of the processes and mechanisms involved in the formation of these structures (especially when it comes to composite or hybrid materials) remain unexplored.

This work aims to identify the features of the molecular characteristics of collagens of different species origin (bovine and marine) and the changes they undergo during enzymatic hydrolysis by pancreatin and thrombin during scaffold formation, as well as the features of the supramolecular structures in the composition of the hybrid hydrogel scaffolds resulting from their combination with fibrinogen.

## 2. Results and Discussion

### 2.1. Characteristics of Native Collagen—CC and BC

As evidenced by data in the literature, the characteristics of native collagen isolated from the tissues of various animal species differ significantly in several parameters: molecular weight characteristics, amino acid ratios and sequences, as well as in their physicochemical properties [50,51,52,53]. Taking into account the purpose of this study, in comparable conditions, we investigated native collagen of the first type from cod skin isolated according to the author’s method [54] and bovine collagen-reagent by Sigma-Aldrich Company, isolated from calf skin [55]. Cod collagen and bovine collagen were isolated under comparable conditions by extraction with a solution of acetic acid [54,56]. The retention of the native protein structure for both cod collagen and bovine collagen is proved by the data of their molecular weight parameters. They are presented in Table 1.

**Figure 1:**
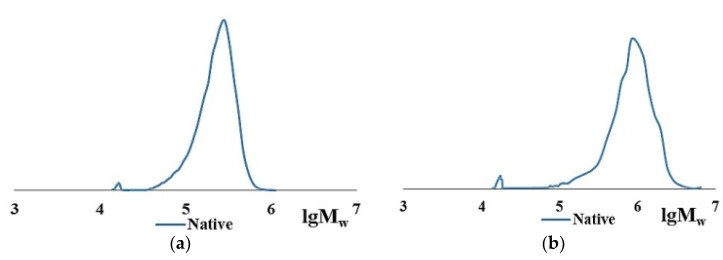
MWD curve of the initial samples of CC (**a**) and BC (**b**).

The main collagen fraction is high molecular weight for both cod collagen and bovine collagen. However, the Mw of the original native bovine collagen is several times higher than that of cod collagen. The Mw of cod collagen has a value of ~300 kDa, corresponds to the literature data, and obviously represents a helix of three α-chains, each of which has a molecular mass of ~100 kDa [33,34]. Bovine collagen is identified as an associate of 2–3 collagen macromolecules with MW of ~300 kDa, the formation of which is possible, according to the literature data, in concentrated aqueous collagen solutions due to intermolecular bonds [57,58]. The polydispersity coefficients in the case of bovine collagen are higher than in the case of cod collagen (Table 1, lines 1, 4). Moreover, in native collagen of both cod and bovine collagen, small amounts of low-molecular-weight fraction with Mw of ~17–20 kDa and traces of a polymer with Mw of ~9–10 kDa appear. Apparently, these are products of partial hydrolysis of native collagen. Close values of hydrolysates according to Mw values when analyzed by the GPC method draw attention to themselves (Table 1). The MWD curves, according to the GPC data of cod collagen (Figure 1a) and bovine collagen (Figure 1b), have an identical form. According to the goal of this study, it is important that both initial proteins are dissolved in aqueous 0.5–1.0 M acetic acid. Such solutions are the starting point for experiments.

**Figure 2:**
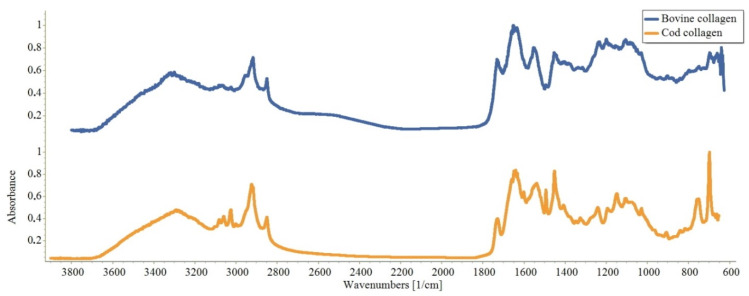
IR spectra of CC and BC films.

The high degree of similarity of the proteins is also evidenced by the IR spectra of the CC and BC films (Figure 2)—they are practically the same. They have absorption bands characteristic of proteins, corresponding to oscillations in the following ranges: 1600–1700 cm^−1^—NH- and C=O-bonds; 1510–1570 cm^−1^—plane bending vibrations of NH-bonds; 1200–1350 cm^−1^—bending vibrations of C-N and NH-bonds; 1720–1730 cm^−1^—stretch vibrations of the carboxyl group, C=O.

### 2.2. Characteristics of the CC and BC Hydrolysis Products

A comparative analysis of the molecular weight characteristics of the CC and BC hydrolysis products formed in the presence of the proteolytic enzymes pancreatin and thrombin was carried out at room temperature. The results indicate similarities and differences for the hydrolysis process occurring under comparable conditions for the specified substrates. The majority of the high molecular BC and CC macromolecules at room temperature are destroyed within the first minute in the presence of both thrombin and pancreatin. However, there are differences in the amounts of the low molecular fractions and the behavior of the initial CC and BC during the process of hydrolysis (data on hydrolysis in the presence of thrombin are shown in Table 2).

It may be recalled that in the process of enzymatic hydrolysis of collagen, both by denaturation via the destruction of the triple-stranded structure and by the destruction of individual chains to low-molecular-weight peptides and oligomeric peptides can occur [59]. The CC and BC hydrolysates consist of several fractions, two of them with close Mw values and a polydispersity coefficient close to 1.0: most of them have Mw ~ 9–10 kDa (80–90%), while smaller proportions have Mw ~ 17–20 kDa (less than 10%).

**Figure 3:**
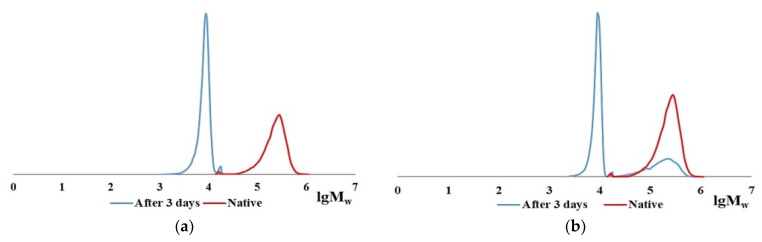

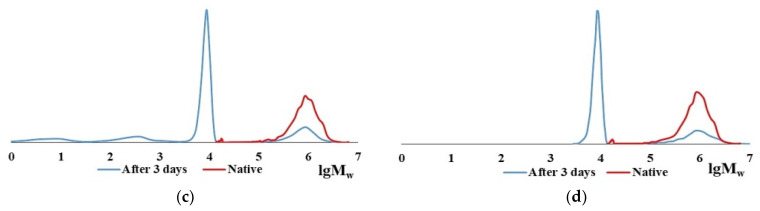
MWD curves of CC hydrolysates with pancreatin (**a**) and thrombin (**b**), BC with pancreatin (**c**), and thrombin (**d**) for 3 days.

**Figure 4:**
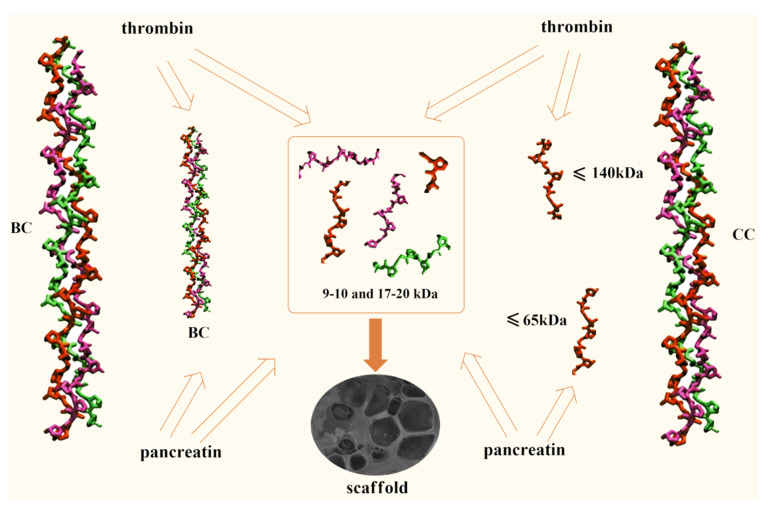
Collagen hydrolysis scheme in the presence of enzymes.

The CC digest solution contains no initial polymer with Mw ~ 300 kDa but does contain macromolecules with Mw ~ 100 kDa [60]. This Mw corresponds to the length of one α-chain; apparently, these are the products of the CC denaturation [33,34]. In the case of BC hydrolysis, ≤10% of the initial high-molecular-weight fraction remains during enzymic hydrolysis in all samples even after prolonged contact with the enzyme for 3 days (Table 2, line 4), while no denaturation products are left. Figure 3 shows the MWD curve dynamics of the enzymatic hydrolysis products of CC and BC over 3 days, from which it is obvious that there is a general tendency for the reduction of collagen macromolecules to low molecular fractions with Mw ~ 9–10 kDa and ~17–20 kDa for both types of collagen, with each of the two different proteolytic enzymes. The hydrolysis products of CC and BC are shown schematically in Figure 4.

Note, particularly, that CC and BC hydrolysates (Figure 3), as well as the fibrinogen hydrolysate [61], have fractions with practically the same Mw values, namely ~9–10 kDa and ~17–20 kDa. This is crucial to define the scaffold formation process. Apparently, due to the fact that proteolytic enzymes hydrolyze the peptide bond formed by the amino acid residues of arginine and lysine [62,63,64], the Mw of the hydrolysates have similar values. It has been suggested [61] that the formation of a hybrid scaffold upon hydrolysis of a mixture of Fg and collagen by thrombin occurs through the fusion of the collagen hydrolysate fibers during the aggregation of the fibrin monomer into the network structure of its polymer [65], thus forming the spatial structure of the scaffold. In the process of enzymatic hydrolysis, the intrinsic viscosity of the collagen solution according to the Mark–Houwink equation (Equation (1)) changes for bovine collagen and cod collagen from 1.03 and 2.35 values up to 0.09 values (the Mw was calculated for the hydrolyzate with Mw ~ 10 kDa, the predominant content of which takes place in both cases). This leads to an increase in the mobility of collagen macromolecules in solution and the formation of the spatial organization of the copolymer.

(1) $$\left[\eta \right]{=KM}^{\alpha }$$

where *K* and *α* are the individual constants of the polymer (for collagen, the values of *K* = 1.34 × 10^−4^, *α* = 0.71) [66,67].

The data obtained in this work are consistent with this hypothesis.

**Figure 5:**
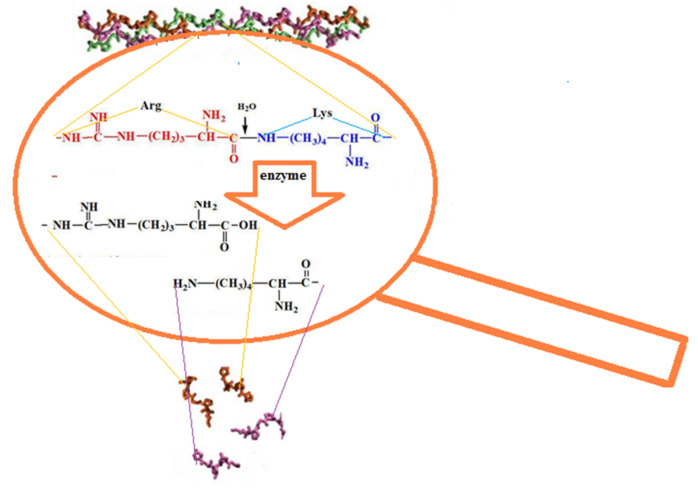
Scheme showing collagen hydrolysate formation—scaffold fragments.

A schematic representation of the enzymatic hydrolysis of collagen is shown in Figure 5.

### 2.3. The Role of Collagen Hydrolysates in the Formation of the Spatial Structure of Scaffolds

**Figure 6:**
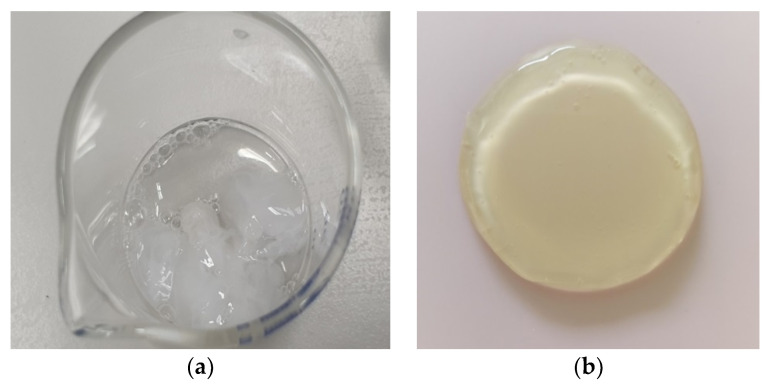
Photographs of a coagulate from a hydrolysate of a CC and Fg mixture (**a**) and of a CC and Fg scaffold (**b**).

In order to clarify the picture of the supramolecular design of scaffolds, joint hydrolysis by thrombin of a 1:1 ratio of Fg and collagen at a high protein concentration (about 1%) was carried out. After 10 min, a coagulate had formed in the solution (Figure 6a) that was analogous to a hybrid scaffold obtained from collagen and fibrinogen (Figure 6b).

**Figure 7:**
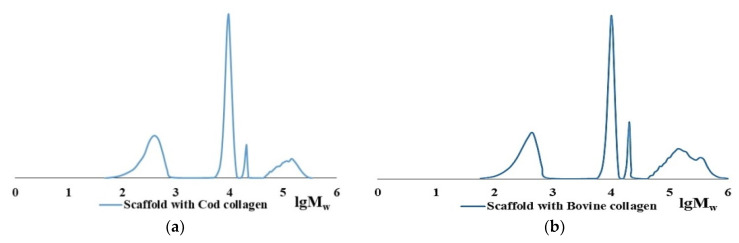
MWD curves of polymer fractions of hybrid scaffold hydrolysates based on fibrinogen with collagen: CC (**a**) and BC (**b**).

It has previously been noted that a water-insoluble fibrin-polymer can be dissolved in dilute solutions of urea or acetic acid [65]. We were able to dissolve hybrid hydrogel scaffolds, based on both CC and BC combined with Fg and obtained according to Section 3.3, in 3% acetic acid over a period of 5 days. Analysis of these solutions using both GPC and dynamic light scattering indicated the destruction of the high molecular structure. According to the GPC data, four fractions were present in the solutions (Figure 7). The two low molecular fractions had Mw values of ~10 kDa and ~20 kDa (M_w_/M_n_ ~ 1.0). The high molecular fraction had Mw values less than 150 kDa with M_w_/M_n_ = 1.2–1.3 for CC, while that derived from BC was less than 240 kDa with M_w_/M_n_ = 1.4–1.6 (Table 3). The contents of the first three fractions for BC and CC are comparable. The high molecular fraction of the scaffold hydrolysate with BC is of greater importance in comparison with that for CC, the value of the polydispersity coefficient and its content being much higher. The rather high polydispersity of the high molecular fractions, especially in the case of BC, indicates their heterogeneity in terms of Mw. The latter suggests that this is an incompletely hydrolyzed high molecular polymer.

**Figure 8:**
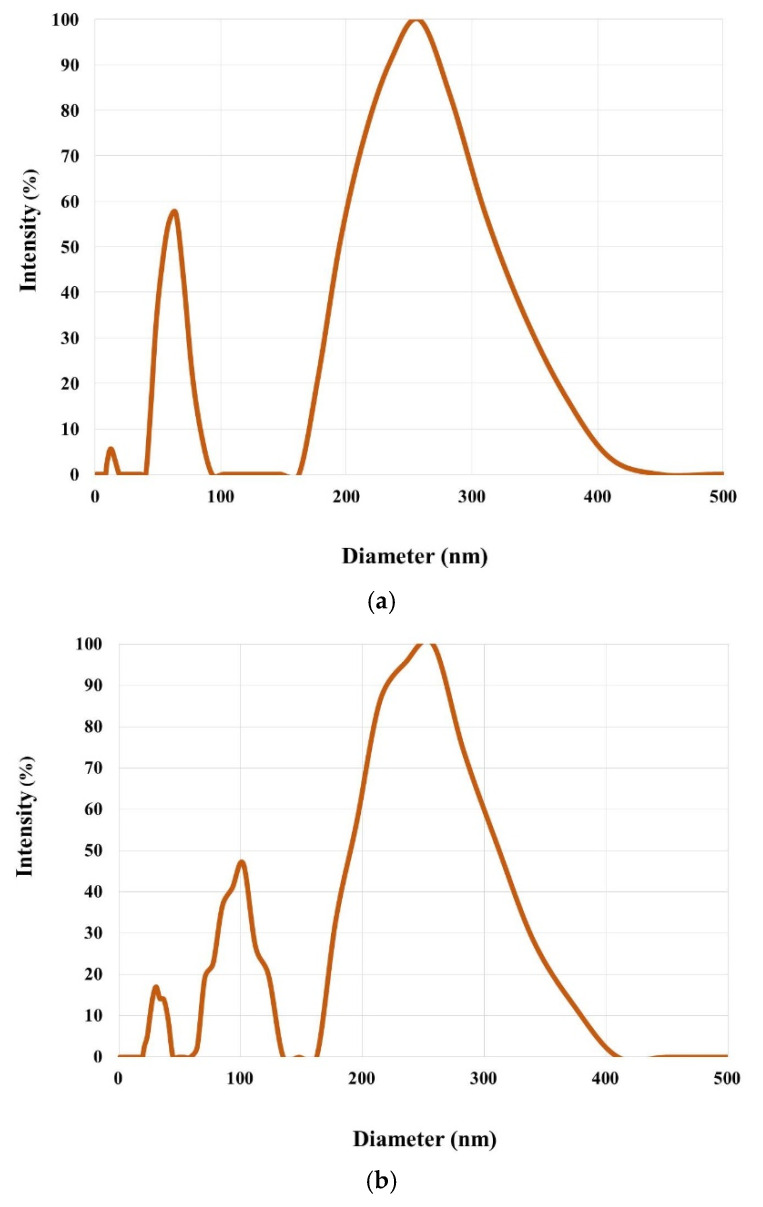
Particle size distribution curves for the scattering intensity of solutions of scaffolds with CC (**a**) and BC (**b**) in acetic acid solution.

The particle size distribution curves for the scattering intensity of acetic acid solutions of scaffolds with CC (a) and BC (b) (Figure 8) are very similar to each other. The method allows us to specify three groups of macro coils of sizes up to ~50 nm, ~50–150 nm, and ~250–500 nm. Accordingly, the first group can be attributed to individual macromolecules, which were recorded using GPC, with Mw up to 150 kDa. The other two groups are associations of macromolecules peculiar to such concentrated aqueous solutions of polar monomers [57] that include natural proteins.

### 2.4. Features of the Supramolecular Structure of Structure-Forming Biopolymers and Scaffolds Produced from Them

**Figure 9:**
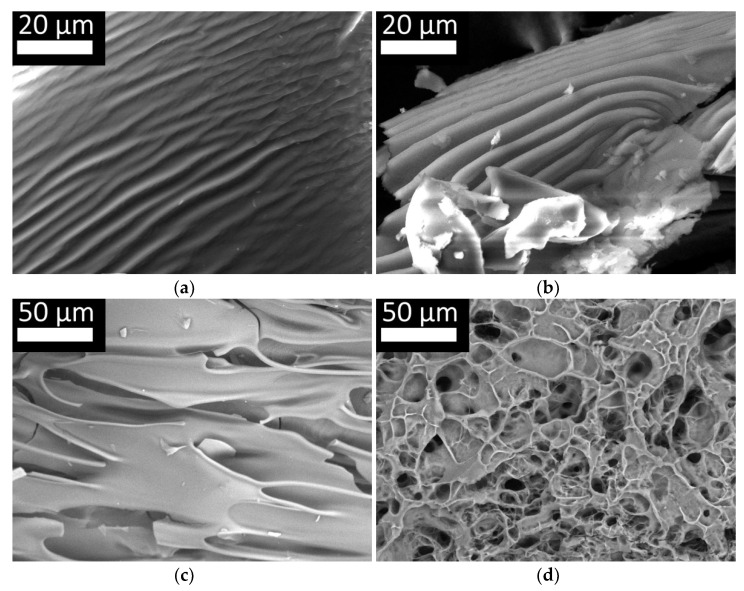
Microstructure of native CC (**a**), BC (**b**) and fibrinogen (**c**) lyophilisate film samples; structure of the coagulate based on Fg and CC (**d**)—lyophilisate, cross section.

Scanning electron microscopy was used to analyze the supramolecular structures of CC and BC scaffolds formed in association with Fg. Figure 9 shows samples of films of the initial CC (a), BC (b), Fg (c), and the structure of the flocculants based on Fg with CC (d). The photographs show that both types of collagen have a fibrillar structure, represented by lengthwise stretched fibers. In the process of preparation and formation of collagen films (in the process of water evaporation, a discrepancy between the rate of water evaporation, the density of the material for research, etc.), the samples of different morphology were obtained. However, threadlike structural homogeneity of both cod collagen and bovine collagen draws attention to itself. Native Fg, unlike collagen, does not have a fibrillar structure and is present in the form of plates with uneven edges. The picture of the structure of the coagulate obtained by the combined hydrolysis of CC and Fg is in marked contrast (Figure 9d). It is characterized by developed reticular microarchitectonics with a system of heterogeneous pores. The unity of the structure is noteworthy. It is impossible to distinguish the Fg or collagen fibers, indicating the formation of uniform structural elements from fibrin and collagen hydrolysates as a result of the enzymatic hydrolysis.

**Figure 10:**
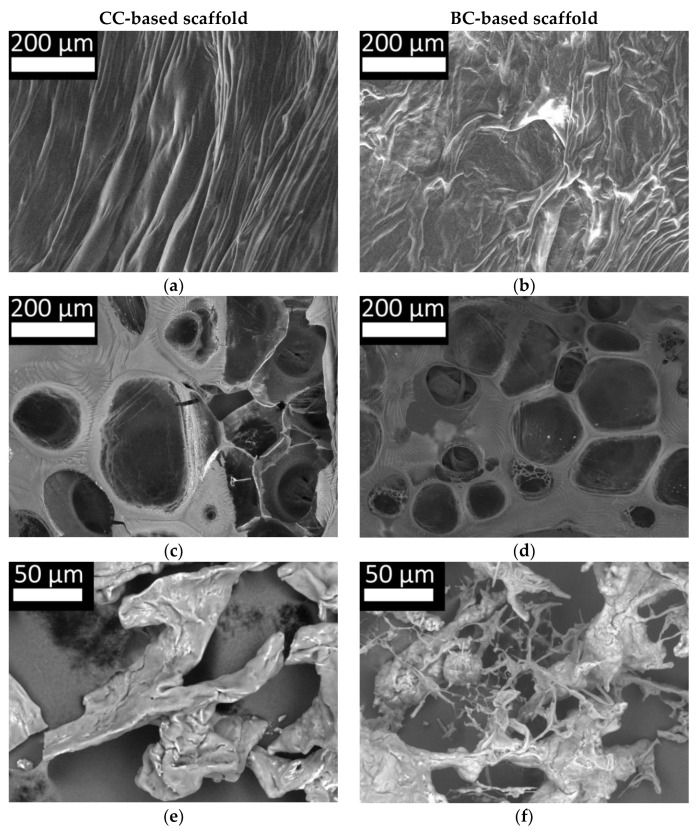
Microstructure of hybrid scaffold samples based on collagen and Fg: microarchitectonics of the scaffold surface (**a**,**b**), the internal architectonics of scaffolds seen in cross section—dehydrated under low vacuum conditions (**c**,**d**); the structure of scaffolds dissolved in 3% acetic acid—lyophilisates (**e**,**f**).

At the next stage, we studied the structure of hybrid hydrogel scaffolds based on Fg with CC (Figure 10a,c) and BC (Figure 10b,d) obtained through the enzymatic hydrolysis reaction, as well as of solutions of these scaffolds in 3% acetic acid (Figure 10e,f), respectively, for CC and BC). The surface structure of the scaffolds (Figure 10a,b) was characterized by the presence of closely spaced fibers and resembled the fibrillar structure of native collagen (Figure 9a,b). However, it should be noted that the surface structure of the CC and BC scaffolds was not identical. The fibers of scaffolds with CC were stretched lengthwise and tangled with each other longitudinally. The fibers of the BC scaffolds were also oriented lengthwise. However, unlike the fibers of the CC scaffolds, the fibers of the BC scaffolds were “branched” and tangled in both the longitudinal and transverse directions. It should be noted that during the scaffold formation, all components and production environments were identical, except for the type of collagen. Thus, differences in the collagen, or rather in the products of its hydrolysis, must have determined the differences in the structural characteristics of the scaffolds. This was despite the amount of fibrinogen in the composite for preparing the scaffolds being 22 times higher than the amount of collagen. Thus, even with such an unequal ratio, collagen plays an essential role in the formation of the structural elements of such scaffolds. This is consistent with our earlier data, which showed differences in scaffold densities and their elastic properties [44]. Differences in internal architectonics were also discussed, so we shall now focus on discussing the features of the internal structure of the scaffolds compared with the structure of the coagulate obtained by the combined hydrolysis of Fg and collagen.

The structure of the hybrid scaffolds (Figure 10c,d) was different from the structure of the coagulate (Figure 9d). Thus, when comparing the structural elements, it can be noted that the membranes between the pores in the scaffolds are thicker and that pores of a much larger size can be observed. These differences could be due to three factors. The first is the different ratio of concentrations of the main structure-forming proteins in the scaffolds and coagulate. In the coagulate, the ratio of Fg and collagen was 1:1, while in the scaffolds, it was 22:1, respectively. The second is the difference in the composites. The coagulate composite consisted of a solution of “pure” Fg and “pure” collagen. The composite for the formation of scaffolds also contained “pure” collagen, but the fibrinogen was in the form of blood plasma cryoprecipitate. It is known that blood plasma cryoprecipitate contains not only fibrinogen but also other proteins, for example, factor XIII, fibronectin, fibrinolysis inhibitors, and cell adhesion molecules [68,69]. According to the literature, these proteins can also take part in the formation of the scaffold structure by forming bonds with the fibrin matrix [70,71,72]. The third factor is the PEGylation of the protein. For the methodology used to produce the hybrid scaffolds studied here, the proteins present in blood plasma cryoprecipitate, including the fibrinogen, are exposed to PEG, which, according to the literature, can lead to cross-linking of the protein molecules [73,74]. However, despite these factors and the revealed differences, the general picture is that the hybrid hydrogel scaffold and the structure of the coagulate had much in common. Thus, the internal architectonics of the scaffolds (Figure 10c,d) was represented by a three-dimensional structure with a heterogeneous pore system and was similar to the three-dimensional structure of the coagulate obtained by the combined hydrolysis of Fg and collagen (Figure 9d). It was impossible to distinguish individual fibrin or collagen fibers in the study using scanning electron microscopy of the dehydrated sections of scaffold samples, as in the case of the coagulates. Thus, we can conclude that the formation of the structural elements of hybrid scaffolds based on Fg and collagen as a result of enzymatic hydrolysis is principally due to the interaction of the hydrolysates of the main structure-forming proteins. They determine the presence of a three-dimensional structure with heterogeneous porosity. At the same time, differences regarding the fiber thicknesses, their interlacing, the size of the walls between the pores, the sizes of the pores, etc., can be due to various factors, including the origin of the collagen and the characteristics of its hydrolysates. The latter is confirmed by the results of the study of the polymer structures and the freeze-dried solutions of scaffolds in 3% acetic acid. The images for scaffolds with CC (Figure 10e) and scaffolds with BC (Figure 10f) show differences in their microstructure. Both samples, with either cod or bovine collagen, have fragments of interconnected fibers, although these are less extensive than in the initial collagen. However, the sample with CC presents only located fibrillar areas, while the sample with BC showed narrow, short fibrils in its structure. Most likely, these fibrils are composed of the unbound fibers of high molecular BC that were found to be present in hydrolysates using the GPC method (Table 1 and Table 3, Figure 3 and Figure 7). They have Mw greater than those of CC and are distinguished by their different structure.

## 3. Materials and Methods

### 3.1. Enzymatic Hydrolysis of Collagen of Various Origins

Pancreatin (Hubei Maxpharm Industries Co, LTD, Wuhan, China) of proteolytic activity 2 U/mg, and thrombin (Renam NPO, Moscow, Russia) of proteolytic activity 2 U/mg, were used as the enzymes for the hydrolysis of both the bovine and cod collagen at 25 °C, in aqueous solution at pH ~ 7.0. For the hydrolysis, a 1% solution of each type of high molecular collagen (HMC) was prepared. Then 1M NaOH was added to the 1% HMC solution to neutralize any acid, with the solution subsequently brought to the required volume with distilled water.

The hydrolysis was carried out by adding the relevant enzyme to the resulting mixture at a mass ratio of collagen: enzyme of 10^2^:1. Samples (1 mL) were taken from the reaction medium at regular intervals after the addition of the enzyme. For these, the hydrolysis was interrupted by adding a 4% acetic acid solution (1 mL) to the samples [2,44].

### 3.2. Combined Hydrolysis of CC and Fg by Thrombin

Enzymatic hydrolysis was carried out using thrombin (Renam NPO, (Russia)) at a protein:thrombin ratio of 10^3^:1 at 25 °C, in aqueous solution at pH ~ 7.0. For hydrolysis, a 1% aqueous solution of the relevant protein was prepared. Then, 1 mL samples were taken at certain intervals after the addition of the enzyme. The hydrolysis was interrupted by adding a 4% acetic acid solution (1 mL) to the samples.

### 3.3. Formation of Hybrid Hydrogel Scaffolds Based on Fg and Collagen

Human blood plasma cryoprecipitate obtained from the GBUZ NO blood center (Nizhny Novgorod, Russia) was used as a source of fibrinogen. The protein was PEGylated (PEG-NHS; Sigma-Aldrich, Darmstadt, Germany). Then a solution of 2% collagen was added—either CC isolated from cod skin [54] or BA—bovine collagen (Sigma-Aldrich, Germany). Phosphate buffer (PBS) was added to the resulting mixture at a 7:1 ratio. To polymerize the mixture, a solution of human thrombin (80 U/mL; Sigma-Aldrich, Germany) in 1% CaCL_2_ solution was introduced into it. Studies with the scaffolds were carried out 24 h after their formation. For this, the newly formed scaffolds were flushed with 5 mL of PBS and incubated in a CO_2_ incubator [45,75].

### 3.4. Freeze Drying

Samples of aqueous solutions for analysis on an electron microscope were placed in a container, frozen with liquid nitrogen, connected to a vacuum unit, and with a decrease in pressure of 0.5–1.0 mbar the water was sublimated into a trap while maintaining a negative temperature of the mixture for at least 4 h.

### 3.5. Scaffold Dissolution

Scaffolds were dissolved at room temperature in 15 mL of a 3% aqueous solution of acetic acid for 3 days.

### 3.6. Reading IR Spectra of CC and BC (See Article in “Inorganic”)

An IRPrestige-21 (Shimadzu, Kyoto, Japan) spectrophotometer was used to record the absorption spectra. Range of wave numbers: 500–550 cm^−1^. Accuracy: ±0.05 cm^−1^. Reflecting KBr plates were used to prepare the films.

### 3.7. Determination of the Molecular Weight Characteristics of Proteins Using Gel-Penetrating Chromatography (GPC)

To identify the molecular weight characteristics of the proteins in aqueous solution, a high-performance liquid chromatograph—Shimadzu CTO20A/20AC (Shimadzu, Kyoto, Japan)—with an LC-Solutions-GPC software module was used. Columns—Tosoh Bioscience TSKgel G3000SWxl—with a pore diameter of 5 μm were used for the separation. We used an ELSD-LT II low-temperature light-scattering detector. The flow rate was 0.8 mL/min, with the eluent being a 0.5 M acetic acid solution. Calibration was performed using narrow disperse dextran samples (molecular mass range: 1000–410,000 Da). Before measurements, the solution was filtered through a polyethersulfone filter with a pore size of 0.45 μm.

### 3.8. Scanning Electron Microscopy

To determine the structural characteristics of the collagen, fibrinogen, and scaffolds, a JSM-IT300 scanning electron microscope (JEOL Ltd., Tokyo, Japan) was used. The diameter of the electron probe was 5 nm, and the operating voltage was 20 kV. In order to avoid charging the samples, detectors of low-energy secondary electrons and backscattered electrons were used in low vacuum mode.

### 3.9. Dynamic Light Scattering Method

The average hydrodynamic weight diameters of the scaffold particles with CC and BC in acetic acid solution were determined using dynamic light scattering (DLS) in the polymodal analysis mode of the correlation function. The measurements were carried out at 25 ± 0.1 °C at an angle of 90° over a range from 0.1 to 5000 nm in 1 cm polystyrene cuvettes using a NanoBrook Omni spectrometer (Brookhaven Instruments, NY, Holtsville, NY, USA). The accumulation time of the correlation function was 180 s. The hydrodynamic weight diameter was calculated as the average of 10 parallel measurements. Before measurements were recorded, the cuvette was dedusted three times by rinsing with distilled water filtered through a polyethersulfone filter with a pore size of 0.2 μm.

The samples for measurement were also filtered through a polyethersulfone filter with a pore size of 0.2 μm.

## 4. Conclusions

Based on the studies described, it can be stated that the CC and BC materials used have different initial molecular weight parameters. In the process of hydrolysis, during the first minute under standard conditions, the majority of each of the two types of protein (>80%) was hydrolyzed by the proteolytic enzymes pancreatin or thrombin (collagen:pancreatin ratio = 10:1). In the subsequent period of monitoring of the molecular weight parameters, the values for the BC samples underwent little further change. In the case of CC, the hydrolysis process continued until there had been a complete disappearance of the high molecular fraction. It was shown that all the collagen samples, regardless of the nature of the initial substrate, were hydrolyzed with the formation of two low molecular fractions, either of Mw ~ 10 kDa or ~20 kDa, with the main part of the hydrolysate (more than 80%) falling within the fraction with an Mw of less than 10 kDa, and this tendency could be observed throughout the entire process. It was found that the concentration of the enzyme had practically no effect on the molecular weight parameters of the final products of the protein degradation. Based on the scanning electron microscopy results, it can be concluded that there are differences in the microstructure of the scaffolds containing collagen from the different sources. Thus, the nature of the collagen source determines its molecular weight characteristics as well as those of its hydrolysates. The latter, in turn, mediate the formation of the structural elements and directly impact the microarchitectonics of the scaffolds formed during enzymatic hydrolysis. We anticipate that the data from our study will, in the future, make it possible both to predict the structural characteristics of scaffolds at the production stage and to offer the opportunity to modulate their properties. Of course, this requires further wide-ranging studies.

## Figures and Tables

**Table 1:** Molecular weight parameters * of the high molecular collagen (HMC).

Item No.	Nature of Collagen	Mw × 10^−3^, kDa	Mw/Mn	Content of HMC in the Dry Residue of Collagen, %
1	CC	250–300	1.2–1.3	95–97
2		17–20	1.1–1.2	3–5
3		9–10	1.1–1.2	trace amounts
4	BC	600–950	1.2–1.6	92–93
5		17–20	1.1–1.2	7–8
6		9–10	1.1–1.2	trace amounts

Note: Mw—average molecular weight, Mw/Mn—coefficient of polydispersion. * Results obtained for a series of samples.

**Table 2:** Molecular weight parameters of the initial and thrombin hydrolyzed CC and BC samples.

Item No.	Sample	Parameter Values during Hydrolysis							
		Initial		Min				3 Days	
				1		60			
		Mw_,_ kDa		Mw, kDa		Mw, kDa		Mw, kDa	
		Value	Fraction, %	Value	Fraction, %	Value	Fraction, %	Value	Fraction, %
1	CC	300	96	125–127	15	124	16	121	16
2		17–18	4	17–18	2	17–18	2	17–18	8
3		-	-	9–10	83	9–10	82	9–10	76
4	BC	950	93	990	7	980	8	˃10^3^	10
5		17	7–8	17	5	17	1–2	17	3
6		-	-	9–10	88	9–10	90–91	9–10	87

Note: collagen:thrombin ratio = 10^2^:1.

**Table 3:** Molecular weight parameters of scaffold hydrolysates based on fibrinogen and collagens of different origins.

Item No.	Scaffold Based on	Mw × 10^−3^, kDa	Mw/Mn	Content, %
1	CC	120–150	1.2–1.3	15
		20	1.0	12
		10	1.0	58
		oligomers	1.2	15
2	BC	200–240	1.4–1.6	30
		20	1.0	14
		10	1.0	46
		oligomers	1.2	10

## Data Availability

All data supporting the conclusions of this article are included in this article.
